# Identification of Proteoforms Related to *Nelumbo nucifera* Flower Petaloid Through Proteogenomic Strategy

**DOI:** 10.3390/proteomes13010004

**Published:** 2025-01-15

**Authors:** Zhongyuan Lin, Jiantao Shu, Yu Qin, Dingding Cao, Jiao Deng, Pingfang Yang

**Affiliations:** 1 Marine and Agricultural Biotechnology Laboratory, College of Geography and Oceanography, Minjiang University, Fuzhou 350108, China; q2634500876@163.com (Y.Q.); caodingding@mju.edu.cn (D.C.); 2 State Key Laboratory of Biocatalysis and Enzyme Engineering, School of Life Sciences, Hubei University, Wuhan 430026, China; shujiantao@metware.cn; 3 FAFU-UCR Joint Center for Horticultural Plant Biology and Metabolomics, Fujian Agriculture and Forestry University, Fuzhou 350002, China; 4 Research Center of Buckwheat Industry Technology, School of Life Sciences, Guizhou Normal University, Guiyang 550025, China; djj613@163.com

**Keywords:** *Nelumbo nucifera*, proteogenomics, new proteoforms, petaloidy

## Abstract

*Nelumbo nucifera* is an aquatic plant with a high ornamental value due to its flower. Despite the release of several versions of the lotus genome, its annotation remains inefficient, which makes it difficult to obtain a more comprehensive knowledge when –omic studies are applied to understand the different biological processes. Focusing on the petaloid of the lotus flower, we conducted a comparative proteomic analysis among five major floral organs. The proteogenomic strategy was applied to analyze the mass spectrometry data in order to dig out novel proteoforms that are involved in the petaloids of the lotus flower. The results revealed that a total of 4863 proteins corresponding to novel genes were identified, with 227 containing single amino acid variants (SAAVs), and 72 originating from alternative splicing (AS) genes. In addition, a range of post-translational modifications (PTMs) events were also identified in lotus. Through functional annotation and homology analysis with 24 closely related plant species, we identified five candidate proteins associated with floral organ development, which were not identified by ordinary proteomic analysis. This study not only provides new insights into understanding the mechanism of petaloids in lotus but is also helpful in identifying new proteoforms to improve the annotation of the lotus genome.

## 1. Introduction

Lotus (*Nelumbo nucifera*) is an important aquatic horticultural plant used for park landscaping, which is widely distributed worldwide. The draft genome of lotus was first released in 2013 [1]. Since then, several improved versions of its genome assembly have been released. However, owing to the widely existing short reads and redundant repetitive sequences in its genome, the annotation quality is low; therefore, the accuracy and completeness of the annotation need to be improved. Additionally, due to the limitations of current bioinformatics tools, certain valuable genetic information, such as small open reading frames (sORFs) from the lotus genome draft has often been overlooked. These drawbacks might obstruct important information needed to understand the mechanism underlying different biological processes, especially when –omic techniques are applied.

Using the effective proteomics strategy, a large number of proteoforms can be obtained which leads to the mining of more valuable genetic information [2]. Tissue-specific proteomic studies have shed light on diverse biological processes in plants, ranging from the growth of pollen tubes in *Pyrus bretschneideri* [3], inter-tissue variation in protein-to-mRNA ratio in pollen and seed of *Arabidopsis* [4], the occurrence of post-translational modifications (PTMs) in the flower of *Cannabis sativa* [5], and the flowering time regulation in *Zea mays* [6]. Recent proteomic studies have shown its valuable function in serving as a basis for enhancing genome annotation, which also provides insights into the mechanism underlying important agronomic traits in various plant species, such as *Arabidopsis thaliana* [4], *Oryza sativa* [7], *Medicago truncatula* [8], *Prunus avium* [9], and *Triticum aestivum* [10]. However, to date, there has been limited research on aquatic floral plants.

In this study, we performed an integrated proteogenomic analysis of five different tissues from the *N. nucifera* floral organ for the identification of novel proteoforms involved in floral petaloid and improving its genome annotation. Utilizing nanoflow liquid chromatography combined with tandem mass spectrometry (LC-MS/MS) and RNA sequencing (RNA-seq), we profiled the proteomes and transcriptomes of five distinct lotus floral organs. Furthermore, we identified and documented a multitude of protein posttranslational modifications (PTMs) involved in petaloidy development. Through the discovery of novel genes, single amino acid variants (SAAVs), and alternative splicing (AS) events, the annotation information for *N. nucifera* was enhanced, offering invaluable insights for future lotus breeding research.

## 2. Materials and Methods

### 2.1. Plant Materials

**Figure 1:**
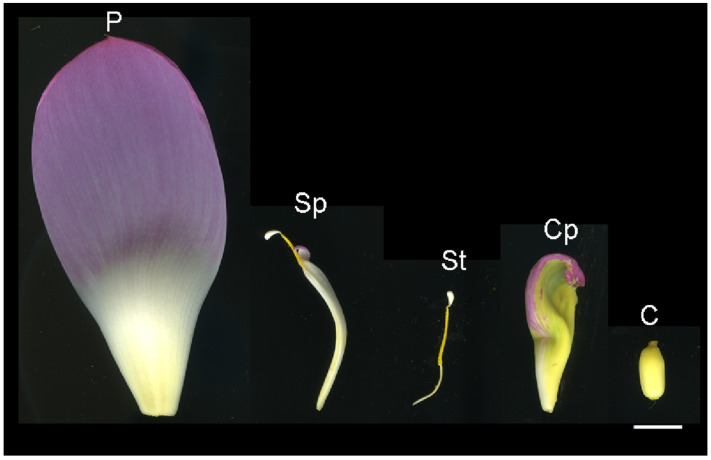
The floral organs of the sacred lotus ‘Sleeping Beauty’. P, petal; Sp, stamen petaoidy; St, stamen; C, carpel; and Cp, carpel petaloidy. The bar indicates 1 cm.

The lotus cultivar “Sleeping Beauty” was cultivated in the Wuhan Botanical Garden, Chinese Academy of Sciences (WBGCAS) in Wuhan, China. Five distinct floral organs, namely Petal (P), stamen petaloidy (Sp), stamen (St), carpel (C), and carpel petaloidy (Cp), were harvested at full bloom (Figure 1). All samples were mixed equally from the three individual lotus. Subsequently, the samples were rapidly frozen in liquid nitrogen and stored at −80 °C until used for protein extraction.

### 2.2. Protein Extraction, Trypsin Digestion, and MS Analysis

Protein extraction was referenced from Deng et al. [11]. Briefly, samples were ground into powder using liquid nitrogen. Then, the powder was thoroughly mixed with pre-cooled homogenate buffer containing 20 mM Tris-HCl (pH 7.5), 1 mM phenylmethylsulfonyl fluoride (PMSF), 250 mM sucrose, 10 mM EGTA, 1 mM dithiothreitol (DTT), and 1% Trition X-100 for 10 min on ice. The mixture was then centrifuged at 4 °C and 20,000× *g* for 10 min to remove the residues. The resulting supernatant was further treated with an equal volume of Tris-phenol (pH 7.8–8.0) for 10 min at 4 °C. The remaining phase underwent a second extraction with homogenate buffer following the same processes. The supernatant was treated with cold acetone containing 0.07% 2-mercaptoethanol (in a 1:3 *v*/*v* ratio) for 2 h at −20 °C. Finally, the resulting precipitate was washed three times with cold acetone, followed by drying. The protein pellets were then re-dissolved in lysis buffer consisting of 7 M urea, 2 M thiourea, 4% (*w*/*v*) CHAPS, 65 mM DTT, and 0.2% (*w*/*v*) Bio-Lyte. Equal amounts of protein from the three independent biological replicates (of each sample) from three different loti were pooled. The protein concentration was determined using the Bradford method [12]. Moreover, SDS-PAGE of total protein preparations was performed (Figure S1). The proteins in the supernatant were stored at −80 °C for further analysis.

The extracted proteins (100 μg of protein per sample) were incubated in 10 mM DTT for 1 h at 56 °C. After cooling to room temperature, proteins were alkylated in 40 mM iodoacetamide for 30 min at 37 °C in the dark. The sample was then diluted with ddH_2_O. Trypsin was added to the sample at a 1:50 trypsin-to-protein ratio and incubated at 37 °C for 16 h on a rocking shaker, followed by a second digestion with a 1:100 trypsin-to-protein mass ratio for an additional 4 h digestion. After trypsin digestion, the samples were centrifuged at 20,000 *× g* for 10 min at 4 °C to separate the supernatant, which was then dried using vacuum centrifugation. The quantified peptide samples were stored at −20 °C until further study.

Each peptide sample was purified and desalted before Nano LC-MS/MS analysis. After digestion, the peptides were reconstituted in a solution containing 5% acetonitrile (ACN) and 0.1% formic acid at a concentration of 1 μg/μL before being loaded into the nano-LC instrument nano ACQuity (Waters, Milford, MA, USA), equipped with the cHiPLC trap (200 μm × 500 μm ChromXP C18-CL, 3 um, 300 Å). A total volume of 5 μL was injected for each analysis. MS analysis was performed using a Nanospray III source and a TripleTOF 5600 plus mass spectrometer (AB SCIEX, Framingham, MA, USA). The MS/MS data were analyzed using the MaxQuant computational proteomics platform [13]. The dataset of lotus proteins utilized for this study was derived from predicted data obtained from lotus genome sequences [1]. Label-free quantitation was conducted using MS/MS signal intensity. The peak intensities of parent peptides were integrated and compared to determine protein expression levels between samples utilizing the Andromeda algorithm. Protein quantification and statistical significance were determined through Student’s *t*-test and error correction with a significance level set at *p* < 0.05 using the Benjamani–Hochberg method. The identified peptides and proteins remained with a false discovery rate (FDR) ≤ 1.0%. Differentially expressed proteins were defined with an absolute fold change value ≥ 1.5 and a *p*-value ≤ 0.05.

### 2.3. Proteogenomic Analysis

Mass spectrometry data generated in this study were deposited in the ProteomeXchange Consortium (http://proteomecentral.proteomexchange.org, accessed on 21 June 2021) with the dataset identifier PXD016222. RNA-seq data of *N. nucifera* were obtained from NCBI (https://www.ncbi.nlm.nih.gov/, accessed on 21 June 2021) using accession numbers PRJNA524054, respectively. Utilizing default parameters, the Trinity tool assembles RNA-seq reads into long transcripts [14]. All raw MS/MS data were transformed into MGF format using the MSConvert tool in ProteoWizard software (version 3.0.4472). And then Comet [15], MS-GF+ [16], and X!Tandem [17] integrated within the GAPE software (https://sourceforge.net/projects/gapeproteogenomic, accessed on 12 January 2025) [18] was performed to search against the six-frame-translated genome database, three-frame-translated genome database, and protein reference database. To address the issue of uneven distribution of false positives in the identified peptides, a target-decoy search strategy was implemented [19]. This strategy involved a stringent filtration approach (≤1% separation) to accurately yield the actual false discovery rate (FDR) for both known and novel peptides. Identified peptides were initially aligned with the predicted protein database using BLASTP. Any peptides that did not match with known proteins were mapped to unique genomic locations and labeled as genome search-specific peptides (GSSPs). These GSSPs were further analyzed to identify new genetic events, such as novel genes, revision of annotated gene models (revised genes), single amino acid variants (SAAVs), and alternative splicing (AS) genes. Novel proteins were reported to contain not less than two unique GSSPs. ORFs that were mapped to regions, not previously annotated, were categorized as novel protein-coding regions, while those that partially overlapped with an annotated gene or exon were designated as gene model revisions. Amino acid mutations or splicing junctions were found to be further investigated according to the remaining significant proportion of GSSPs. To identify SAAVs, GSSPs with a minimum length of 10 amino acids were aligned with the genome, allowing for a maximum of two non-synonymous variants within a GSSP. Subsequently, all generated MS data were processed through the GAPE software (https://sourceforge.net/projects/gapeproteogenomic, accessed on 12 January 2025) for accurate peptide and protein identification [18].

### 2.4. Validation of PTMs

To comprehensively identify all potential PTMs in the dataset, an unrestricted database search was performed using MODa as described previously [20]. All known and potentially unknown types of PTMs were considered, with modifications up to a mass shift of 250 Da allowed for each peptide. Additionally, from the previously defined parameters, a targeted search for specific PTMs was carried out against the protein database through MaxQuant software (v 1.6.0.16) [13].

### 2.5. Bioinformatics Analysis

Functional annotation of novel genes was carried out using Blast2GO (version 5.2) [21]. COG analysis was performed with the eggNOG-mapper [22], and pathways mapping was conducted using the Kyoto Encyclopedia of Genes and Genomes (KEGG) database [23]. Subcellular localization was predicted with DeepLoc-1.0 software (https://services.healthtech.dtu.dk/services/DeepLoc-1.0/, accessed on 12 January 2025) [24]. The blast was used for homology analysis (v 2.11.0+), and the conservation of the identified novel protein was assessed through a reciprocal blast of TBtools (v 0.66834) [25]. The identified novel genes were visualized by Integrative Genomics Viewer (IGV) software 2.0 [26]. Statistical analyses were conducted using custom Python and R statistics programs.

## 3. Results

### 3.1. Overview of the Proteomic Analysis

**Figure 2:**
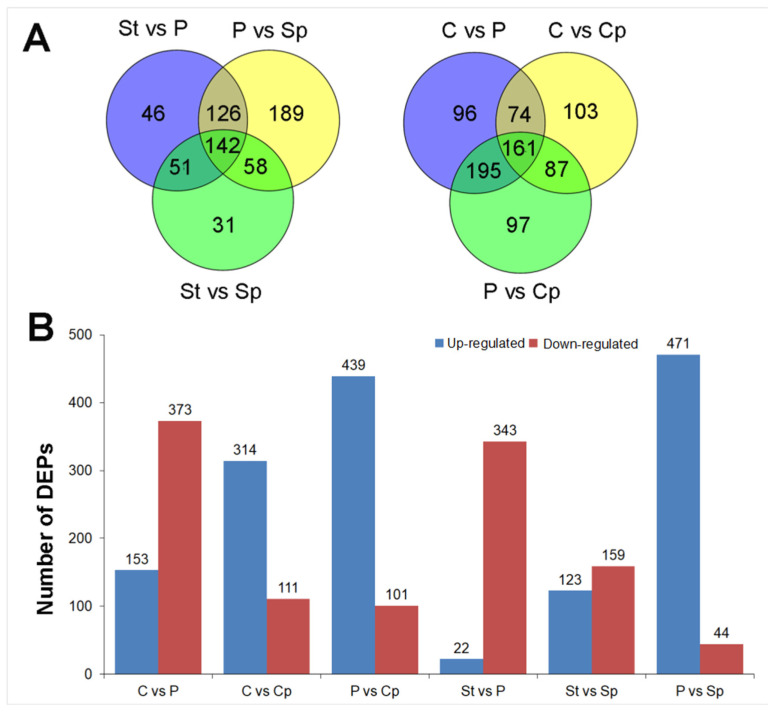
Summary of differentially expressed proteins (DEPs). (**A**) The unique and overlapped DEPs in the comparisons of P vs. Sp, St vs. P, and St vs. Sp; C vs. P, C vs. Cp, and P vs. Cp. (**B**) The number of up-regulated and down-regulated DEPs in each comparison.

In the present study, a label-free quantitative proteomic analysis was used for protein identifications of the lotus floral organs, including P, Sp, St, C, and Cp. A total of 1968 proteins were identified, among which 1561 could be quantified (detected at least twice in three biological replicates) (Table S1). The quantified proteins of P, Sp, St, Cp, and C were 1016, 797, 559, 1057, and 1098, respectively. After filtering for expression fold change greater than 1.50 or less than 0.67 and *p*-value < 0.05, pairwise comparison between P, Sp, and St, revealed 142 common differentially expressed proteins (DEPs, Figure 2A), while comparisons among P, Cp, and C showed 161 common DEPs (Figure 2A). The number of DEPs in various pairwise comparisons was shown as follows: C vs. P (526 proteins), C vs. Cp (425 proteins), P vs. Cp (540 proteins), St vs. P (365 proteins), St vs. Sp (282 proteins), and P vs. Sp (515 proteins). In these comparisons, 153, 314, 439, 22, 123, and 471 proteins were up-regulated DEPs, while 373, 111, 101, 343, 159, and 44 were down-regulated DEPs, respectively (Figure 2B). Among the proteins from P, Cp, and C groups, 161 common DEPs might be more closely related to carpel petaloidy (Figure 2A). Hence, were selected for further GO and KEGG analyses (Figure S2). GO annotation identified 156 proteins, with the most enriched GO terms in biological processes being gluconeogenesis and tricarboxylic acid cycle, the chloroplast stroma in cellular component, and copper ion binding in molecular function. Meanwhile, the most enriched KEGG pathway included carbon metabolism, biosynthesis of amino acids, citrate cycle, pyruvate metabolism, glycolysis/gluconeogenesis, and carbon fixation in photosynthetic organisms.

### 3.2. Comparative Analysis of Transcriptome and Proteome Data

Similarly to the previously reported transcriptome analysis [27], different groups were classified based on the stamen petaloidy group (St vs. P, P vs. Sp, St vs. Sp) and the carpel petaloidy group (C vs. P, C vs. Cp, P vs. Cp). After identifying common elements, a total of 19 cor-DEGs-DEPs were selected, including 12 (St vs. P), 1 (P vs. Sp), and 9 (St vs. Sp) in the stamen petaloidy group (Figure S3A). In the stamen petaloidy group, only a small number of cor-DEGs-DEPs were found in the stamen petaloidy and petal. On the other hand, there were 270 genes identified as cor-DEGs-DEPs in the carpel petaloidy group, including 174 (C vs. P), 75 (C vs. Cp), and 135 (P vs. Cp) (Figure S3B). Furthermore, a comparison of transcriptomic and proteomic data in the carpel petaloidy group led to the identification of several carpel petaloidy-related candidate genes.

**Figure 3:**
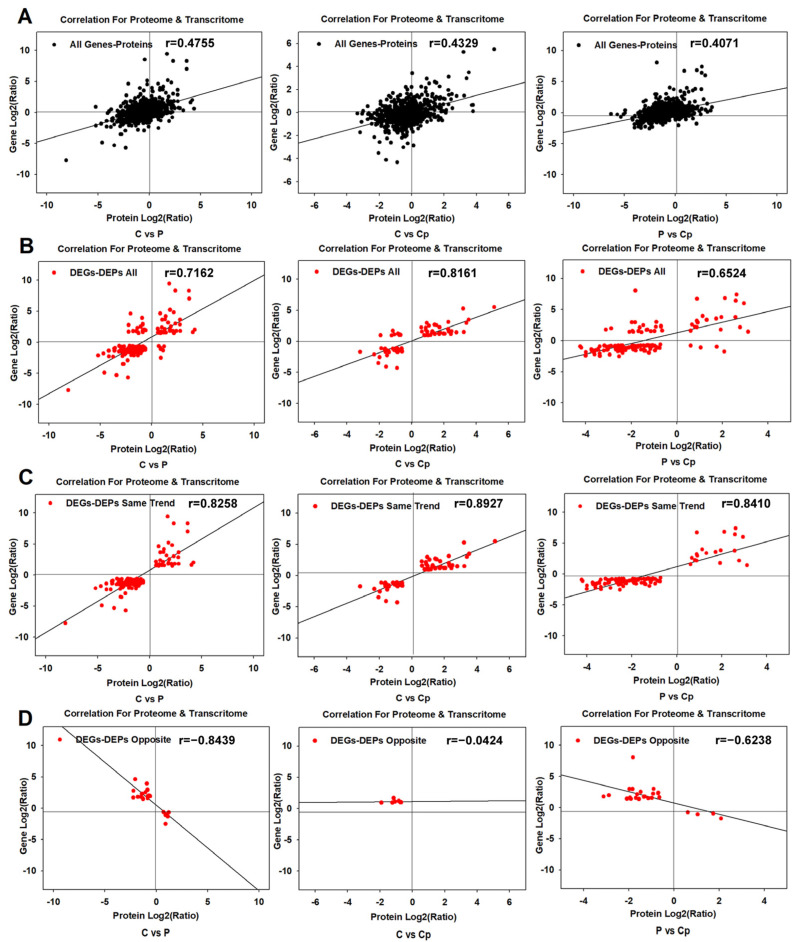
Relationship between transcriptomic and proteomic data. (**A**) Scatterplots depicting the correlation of the relationship between proteomic and transcriptomic datasets. (**B**) Scatterplots and correlation coefficients illustrate the relationship between DEPs and DEGs. Scatterplots and correlation coefficients show the relationship between protein and transcript expression ratios with either similar (**C**) or opposite (**D**) changing trends.

The total number of commonly expressed genes or detected proteins among petal, carpel petaloidy, and carpel were 755 (C vs. P), 827 (C vs. Cp), and 722 (P vs. Cp) in the carpel petaloidy group, respectively. Their Pearson correlation coefficients were 0.4755 (C vs. P), 0.4329 (C vs. Cp), and 0.4071 (P vs. Cp), respectively (Figure 3A). Furthermore, the correlations (Pearson) of cor-DEGs-DEPs were 0.7162 (C vs. P), 0.8161 (C vs. Cp), and 0.6524 (P vs. Cp), indicating that the Pearson relationship of cor-DEGs-DEPs was higher than that of overall expressed genes and proteins (Figure 3B). The consistent trends in the relationship between DEGs and DEPs with high correlation coefficients were 0.8258 (C vs. P), 0.8927 (C vs. Cp), and 0.8410 (P vs. Cp) (Figure 3C), which could be further investigated. Finally, the correlation coefficients representing the opposite trend between DEGs and DEPs in C vs. P, C vs. Cp, and P vs. Cp were −0.8439, −0.0424, and −0.6238, respectively (Figure 3D). Due to the limited number of cor-DEGs-DEPs with opposing trends, the relevance was extremely low in C vs. Cp.

In the carpel petaloidy group, 230 cor-DEGs-DEPs were identified as exhibiting the same tendency, including 147 in the C vs. P comparison, 67 in the C vs. Cp comparison, and 110 in the P vs. Cp comparison (Table S2). Among these genes, 222 genes were annotated, and GO analysis results comprised biological process, cellular component, and molecular function with 44 belonging to the critical functions group (Figure S3A). The cellular process and metabolic process were the two major groups in the biological process. Cell components had two largest groups, including cell part and cell. Molecular function mainly contained binding and catalytic activity groups. In GO enrichment, the biological process principally consisted of tricarboxylic acid cycle and pentose-phosphate shunt; the cellular process majorly contained apoplast; copper ion binding is a primary group in molecular function (Figure S3B). Additionally, KEGG analysis was also performed. A total of 230 cor-DEGs-DEPs were enriched into 77 KEGG pathways (Table S3). The results highlighted the enrichment of KEGG pathways such as carbon metabolism (ko01200), citrate cycle (TCA cycle, ko00020), biosynthesis of amino acids (ko01230), and glycolysis/gluconeogenesis (ko00010). Furthermore, six common cor-DEGs-DEPs were identified in the comparisons between petal, carpel petaloidy, and carpel pairwise comparisons.

### 3.3. Identification of Novel Proteoforms Originated from Novel Genes, Alternative Splicing Events, and Single Amino Acid Variants (SAAVs)

**Figure 4:**
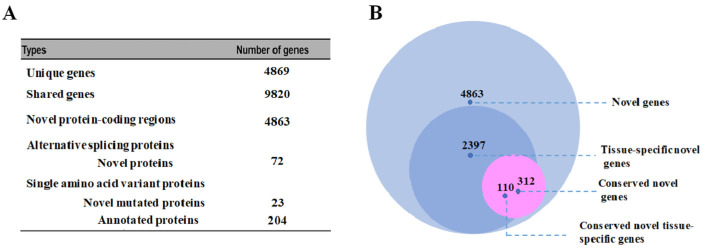
Diagram of the results in GAPE tool identification. (**A**) Overview of the identifications from the present study. (**B**) Total number and overlap of identified gene loci in novel genes, tissue-specific novel genes, conserved novel genes, and conserved novel tissue-specific genes.

To further dig out the MS data, a proteogenomic analysis was conducted using the GAPE tool. The spectral raw data were analyzed with three search engines against a proteogenomic database to identify new peptides. These identified peptides were further matched to known protein databases to differentiate between known peptides and unique orphan peptides. It is worth mentioning that these unique orphan peptides were not previously recognized as proteins in the UniProt database for the lotus species. Furthermore, these unique orphan peptides were then mapped to the lotus genome with the help of BLAST. Peptides that could be specifically located in the genome were named genome search-specific peptides (GSSPs). Subsequently, these GSSPs were subjected to additional genome blasting to potentially uncover new events, such as novel genes, revision of annotated gene models (revised genes), alternative splicing (AS) genes, and single amino acid variants (SAAVs). In our study, a total of 4869 unique genes and 9820 shared genes were identified (Figure 4A). The shared gene is defined as encoding the proteins identified solely through the shared peptides. There were 4863 novel protein-coding regions, 2397 tissue-specific novel genes, 312 conserved novel genes, and 110 conserved novel tissue-specific genes from the known protein sequence of lotus (Figure 4A,B; Table S4A). Furthermore, 72 AS genes and 227 SAAVs were identified (Figure 4A; Table S4B–D). The 227 SAAVs contained 204 annotated proteins and 23 novel mutated proteins (Figure 4A; Table S4C,D). All these indicate a significant number of novel proteoforms.

**Figure 5:**
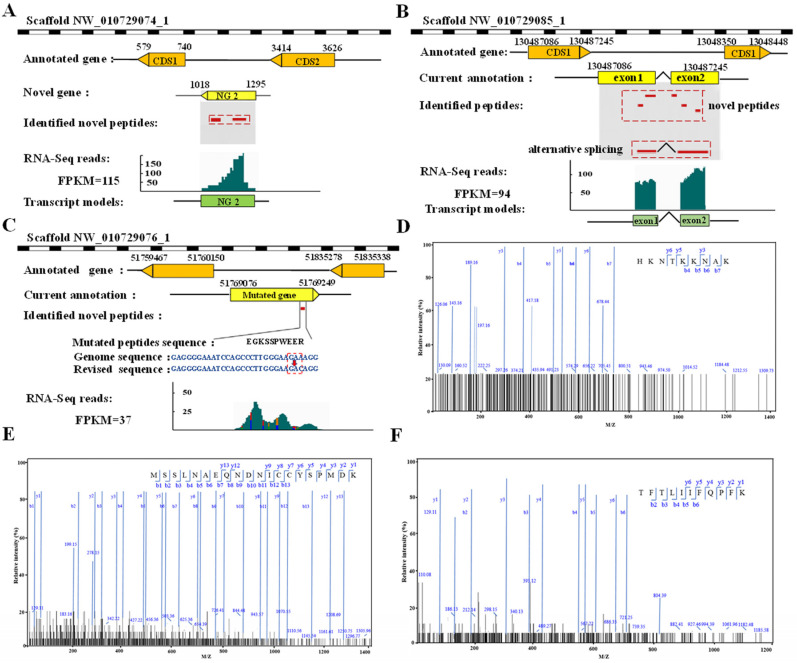
Discovery of novel genes, novel alternative splicing events, and SAAVs. (**A**) Novel peptides located in intergenic regions. Two peptides were pinpointed within a region on scaffold NW_010729074_1 of the *N. nucifera* genome, devoid annotation of genes. (**B**) Detection of novel exons on scaffold NW_010729085_1. A splicing peptide was identified in an intronic region in a new locus. Examination of the transcripts also indicates the presence of a spliced variant for this locus. (**C**) Detection of SNPs on Scaffold NW_010729076_1. An existing gene was unraveled through the identification of one single SNP-containing peptide as well as RNA-seq data. (**D**–**F**) Validation of three novel peptides (comprising a novel gene peptide, a novel splice junction peptide, and a novel SNP-containing peptide) by comparing the MS spectra of the identified peptides from proteogenomic analysis. The MS spectra of the novel peptides (HKNTKKNAK [D], MSSLNAEQNDNICCYSPMDK [E], and TFTLIIFQPFK [F]) are shown.

As Figure 5A shows, the novel gene NG2 contained two unique intergenic peptides that were mapped to a genomic region spanning nucleotides 1018–1295, indicating the presence of a novel protein-encoding gene. The validation of this gene was further supported by our transcriptomic data. Peptides with spanning exon–exon boundaries can aid in the identification of novel splice events. Utilizing five novel peptides located on two exons, a new alternative splicing event was uncovered (Figure 5B), which was also confirmed by the RNA-seq data. The relationship between SAAVs and reliable function-associated genetic variations is crucial. For example, a GSSP mutation with A to C substitution led to the alteration of the glutamic acid codon GAA to the aspartic acid codon GAC (Figure 5C).

To validate the new events at the protein level, we analyzed the novel peptides of NG2, as well as the novel AS and mutate proteins identified by Proteome Discoverer software 2.5. The corresponding MS/MS spectral data of the identified GSSPs was retrieved from our proteogenomic analysis (Figure 5D–F).

### 3.4. Structure and Function Analysis of Novel Proteoforms and Their Corresponding Genes

**Figure 6:**
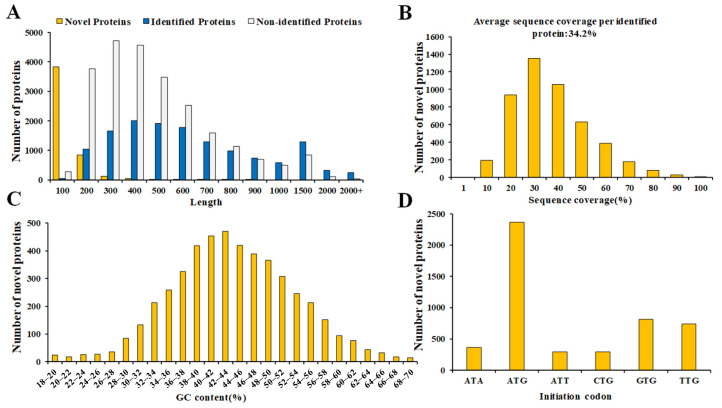
Overview of proteogenomic analysis. (**A**) Bar chart showing the length of all identified proteins in proteogenomic analyses. (**B**) Bar chart showing protein sequence coverage. (**C**) Bar chart showing the GC content of novel proteins via proteogenomic approach. (**D**) Bar chart showing the distribution of translation start codon of the coding genes of identified novel protein via proteogenomic approach.

The majority of novel proteins identified in this study were found to be less than 400 amino acids in length, with an average length of 162 aa (Figure 6A; Table S5A). A comparison with the length of identified annotated proteins revealed a significant difference in the length of unidentified annotated proteins (Table S5B,C), indicating that these were mainly novel proteins encoded by shorter ORFs. The average sequence coverage per identified novel protein was found to be 34.2% (Figure 6B), and GC contents of the most novel identified genes ranged from 20% to 60% (Figure 6C; Table S5A). Analysis of the start codon frequency for the identified novel protein-encoding genes showed that ATG was the most predominant, accounting for over 48.6% of the start codons, followed by GTG and TTG as the second and third most frequent (Figure 6D; Table S5A).

**Figure 7:**
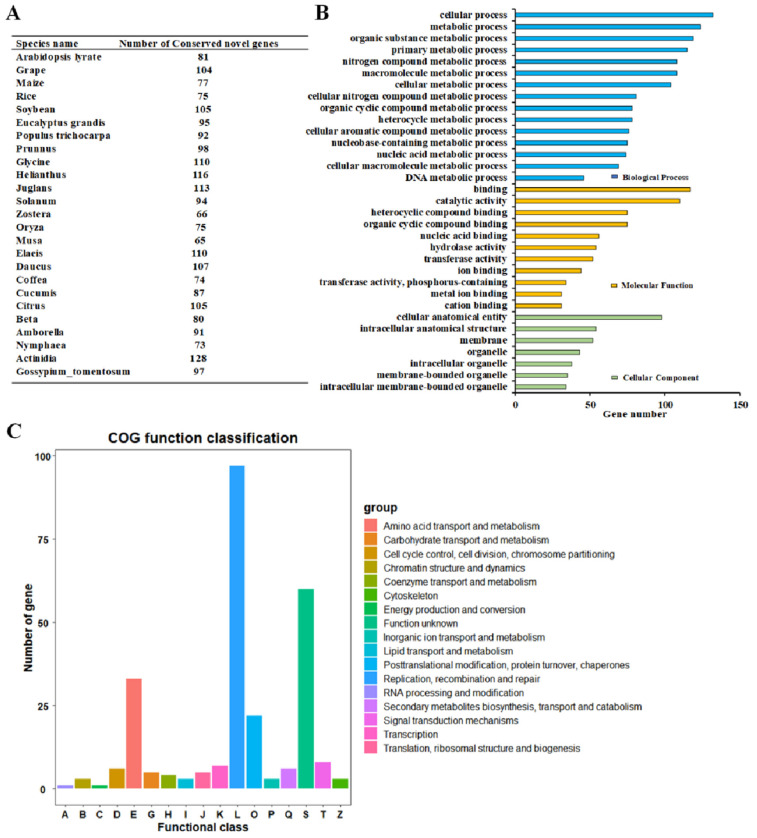
Summary of post-translational modification of identified proteins. (**A**) The number of conserved novel genes in lotus compared to other plants. (**B**) The identified novel genes annotated to involve in GO biological process, molecular function, and cellular localization terms. (**C**) The identified novel genes were classified by COG function.

To further clarify the biological function of these putative novel proteoforms, functional annotation was performed. Subcellular localization analysis of the 4863 identified novel proteins revealed that a significant portion of the proteins (2173, 44.6%) was predicted to be localized in the nucleus, followed by chloroplasts (972, 19.9%) and the cytoplasm (735, 15.1%) (Table S5D). Comparatively, a large percentage of both identified and unidentified proteins were found to be localized in the nucleus, with 42.0% and 41.8%, respectively (Table S5E,F). Similarly, the chloroplast and cytoplasm were the second and third most frequent localization sites for both the identified and unidentified proteins (Table S5E,F). Additionally, a comparison with homologs from 24 other plants revealed that the identified novel proteins were most closely related to *N. nucifera* [28,29]. Among these conserved novel genes, the number of newly identified genes was determined, which are conserved in *A. lyrata*, *Vitis vinifera*, *Z. mays*, *Glycine max*, *Eucalyptus grandis*, *Populus trichocarpa*, *Prunnus persica*, *Glycine soja*, *Helianthus annuus*, *Juglans regia*, *Solanum tuberosum*, *Zostera marina*, *O. sativa*, *Musa nana*, *Elaeis guineensis*, *Daucus carota*, *Coffea arabica*, *Cucumis melo*, *Citrus sinensis*, *Beta vulgaris*, *Amborella trieopoda*, *Nymphaea tetragona*, *Actinidia chinensis*, and *Gossypium tomentosum* (Figure 7A; Table S6A–X). Based on GO classification, a substantial number of identified novel proteins were found to be involved in cellular processes and were associated with binding various targets and cellular anatomical entities (Figure 7B, Table S7A–C). Additionally, functional annotations of these identified novel proteins, based on the NCBI COG, indicated that these proteins could potentially control replication, recombination, and repair processes (Figure 7C; Table S7D).

**Figure 8:**
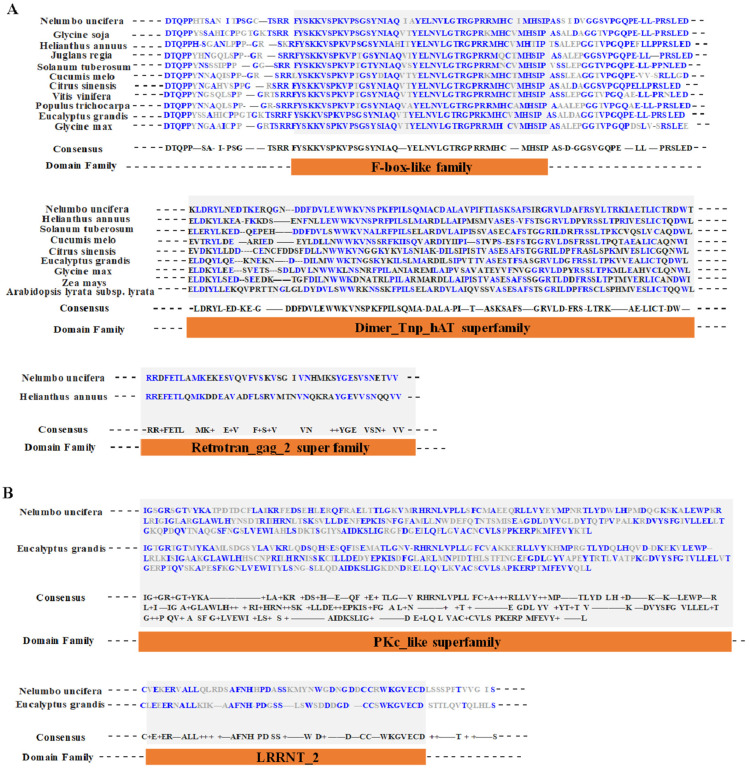
Conserved domain analysis of novel proteins associated with flower organ development. (**A**) Analysis of conserved domains of three novel tissue-specific genes in Cp. (**B**) Analysis of conserved domains of two novel tissue-specific genes in St.

In this study, 110 tissue-specific genes were selected from 312 novel conservation, which might be related to flower organ development (Table S8A,B). Among them, there were 26, 18, 24, 24, and 23 novel tissue-specific genes in C, P, Cp, Sp, and St tissues, respectively (Table S8C–G). As shown in Figure 8, these 110 new genes were then analyzed for their conserved domains and ultimately resulted in the identification of 5 new candidate genes (Table S8H). These five candidate genes have clear functional homologs in closely related species of lotus, with three of them being Cp tissue-specific genes and the other two related to St tissue-specific genes (Table S8H). As illustrated in Figure 8A, one novel candidate protein showed homology with proteins in *Glycine soja*, *H. annuus*, *J. regia*, *S. tuberosum*, *C. melo*, *Citrus sinensis*, *V. vinifera*, *Populus trichocarpa*, *E. grandis*, and *Glyine max*, which contains an F-box-like family domain. F-box-like family members have been known to play a role in floral morphogenesis [30]. The second one had a homologous protein from other plant species—*H. annuus*, *S. tuberosum*, *C. melo*, *C. sinensis*, *E. grandis*, *Glyine max*, *Z. mays*, and *A. lyrata*—which contained a Dimer Tnp hAT superfamily domain (Figure 8A). Additionally, the Retrotran gag 2 superfamily domain was found to be a conserved domain in *N. nucifera* and *H. annuus*, with a significant presence in the floral meristems [31]. As for the novel tissue-specific genes in St, the third and fourth candidate genes were homologous in *E. grandis* with PKc-like family and LRRNT 2 domain-containing proteins of the leucine-rich repeat (LRR) Receptor-Like Ser/Thr Protein Kinase, respectively (Figure 8B). Both of them are protein kinases. PKc-like superfamily proteins, including several receptor kinases, are known to regulate pollen tube growth [32]. On the other hand, RPK2 from *A. thaliana* containing the LRRNT domain is a key regulator of anther development [33].

### 3.5. Discovery of Protein Post-Translational Modification in Lotus Floral Organs

To date, numerous proteomic analyses have been carried out on lotus. However, there has been a lack of studies focusing on protein post-translational modifications (PTMs). In the current study, we identified 3587 potential novel modifications from 1504 proteins by MODa (Table S9A). Because MODa could not resolve the specific amino acid residue where the modifications occurred, MaxQuant software (version 1.6.0.16) was applied to determine the exact site of the modifications. From these PTMs, 18 modifications common in eukaryotes were selected and the localization of modification sites was performed using MaxQuant (Table S9B). We identified 2728 PTMs encompassing 18 different types of modifications (Table S9B). By obtaining a large amount of MS data from five different tissue samples and a large number of PTM results, we have deepened our understanding of PTMs in lotus. The number of carbamyl is the most modifications with 638 (approximate 23.39%). These data provide valuable insights into the diverse array of post-translational modifications occurring in lotus proteins, shedding light on the intricate regulatory mechanisms that govern protein function and cellular processes in this plant species.

## 4. Discussion

In our study, integrated proteomics and proteogenomic analyses of lotus floral tissues were carried out, encompassing 4869 unique genes derived from five major flower organs. Among them, 4863 novel proteoforms containing 72 splice variants, and 227 SAAVs were detected through the presence of specific peptides identified in our MS data using bioinformatic analyses. Moreover, a diverse set of PTMs was comprehensively examined in this study. Through this comprehensive assessment, we pinpointed tissue-specific genes implicated in the development of flower organs. Meanwhile, because of the limitations of proteomic techniques in detecting low-abundant proteins, quantitative analysis on the low-abundant proteins might not be accurate. This would result in a little bit of bias in determining the tissue-specific proteins.

In this study, five floral organs were compared, and their proteoforms were characterized. Meanwhile, transcriptomic data were extracted from a previous transcriptome analysis on the same samples [27]. Based on their complementary analysis, differentially expressed genes associated with petaloidy were identified. From the proteomic analysis data of P, Sp, St, Cp, and C, numerous DEPs related to petaloidy were selected and the expression of total gene and total protein showed poor correlation in association analysis between transcriptome and proteome. This could be attributed to the difference in mRNA transcription and translation expression of the protein. However, there was a strong positive relationship (r > 0.8) in the same trend of cor-DEGs-DEPs (Figure 3) indicating that post-transcription and post-translation potentially happen in the process of RNA and protein expression as previously reported [34,35]. The number of tissue-specific proteins was found to be lower than the number of tissue-specific transcripts, a discrepancy that may be attributed to the significant difference in the total gene numbers observed in the two datasets.

For decades, seed proteomes in lotus have been extensively studied to explore their biochemical and molecular functions, such as the primary metabolism in seed development [36], secondary metabolites in maturing seed plumule [37], seed thermotolerance [38], seed dehydration tolerance [39], seed longevity [40], and identification of antioxidative peptides in seed proteins [41]. Additionally, proteomics on other tissues have also been carried out to identify putative biological functions including the signaling pathway in the rhizome enlargement process [42], floral thermogenesis [43], proteomes of petals involved in pigmentation [11,44], and light signal regulation of shade environment stress [45]. Proteomic studies were carried out to provide more information on the functions of lotus. However, there are still no reports on the proteogenomic analysis of lotus plants.

Here, we performed a proteogenomic study on lotus. The proteogenomic strategy has been utilized to discover new coding events and enhance the original genome annotation database, particularly in the annotation of sORFs [3,18]. A total of 4863 new coding events were identified, with 3847 sORFs containing less than 100 codons (Table S4A). Further investigations are necessary to determine the functions of these novel proteins with small ORFs.

Additionally, our analysis has shown several unique proteins related to the five different flower organs. We found two proteins that were specifically expressed in the stamen and three proteins in carpel petaloidy. Petaloidy is a popular phenomenon in plants. However, the molecular mechanisms of petaloidy remain to be uncovered. In the current study, three proteins involved in carpel petaloidy were found (Figure 8). Especially, a novel tissue-specific protein assigned as an F-box-like family protein showed higher homologous with the other five plant species. BLASTP search was performed in NR and revealed that it mapped tubby-like F-box family protein (XP_019053783.1 with E-value 2e^−173^) with 95% query cover. This protein was consistent with the previous report about comprising a highly conserved F-box domain in addition to the tubby-like protein domain at the C-terminus [46]. Both nuclear transcriptional regulation and plastid subsequently released from the plasma membrane were influenced by tubby-like protein [47]. These findings suggest that tissue-specific proteins may be linked to related biological processes. Thus, proteogenomic analysis is a convenient approach for genome annotation [18,48,49,50]. A comprehensive view of PTM events was exhibited by proteogenomic analysis [3,18,51]. We conducted a systematic analysis of PTM events in lotus to further our understanding. Meanwhile, how these PTMs contribute to flower organ development in *N. nucifera* needs to be further studied with various molecular and biochemistry methods.

## 5. Conclusions

The present study identified a large amount of novel proteoforms through a proteogenomic strategy, which may help to improve the genome annotations of *N. nucifera*. The discovery of novel events and comprehensive protein post-translational modifications (PTMs) in this research offers a significant resource for deeper investigations into the mechanism of flower organ development in lotus. This study not only contributes to a more detailed understanding of the lotus genome but also serves as a valuable template for conducting extensive proteomic studies on other horticultural plants.

## Data Availability

The RNA-seq data generated in this study are available in the NCBI using accession numbers PRJNA524054. The mass spectrometry proteomics data have been deposited to the ProteomeXchange Consortium (http://proteomecentral.proteomexchange.org, accessed on 21 June 2021) via the PRIDE [52] partner repository with the dataset identifier PXD016222.

## Supplementary Materials

The following supporting information can be downloaded at: https://www.mdpi.com/article/10.3390/proteomes13010004/s1, Figure S1: Representative SDS-PAGE of total protein preparations obtained from five different floral organs including petal (P), stamen petaloidy (Sp), stamen (St), carpel (C), and carpel petaloidy (Cp); Figure S2: GO and KEGG analysis of DEPs. (A) GO annotation of DEPs. (B) The most enriched GO terms of annotated DEPs. (C) KEGG classification in DEPs. (D) KEGG enrichment in DEPs; Figure S3: Venn diagram of the number of unique and common DEGs and DEPs. (A)Venn diagram of the number of unique and common in the two comparisons in the P, Sp, and St group (P vs. Sp, St vs. P, and St vs. Sp). (B) Venn diagram of the number of unique and common in the two comparisons in the P, Cp, and C group (C vs. P, C vs. Cp, and P vs. Cp); Figure S4: GO and KEGG analysis of cor-DEGs-DEPs. (A) GO annotation of cor-DEGs-DEPs. (B) The most enriched GO terms of annotated cor-DEGs-DEPs. Table S1: Overview of protein identification; Table S2: Table S2A The same tendency cor-DEGs-DEPs in C vs. P; Table S2B The same tendency cor-DEGs-DEPs in C vs. Cp; Table S2C The same tendency cor-DEGs-DEPs in P vs. Cp; Table S3: KEGG pathway enrichment analysis of the cor-DEGs-DEPs genes. Table S4: Table S4A. List of novel protein-coding genes using genome search specific peptide; Table S4B. List of novel alternative splicing protein-coding genes; Table S4C. List of novel single amino acid variants using genome search specific peptide; Table S4D. List of single amino acid variants from annotated proteins using genome search specific peptide; Table S5: Table S5A. List of novel proteins in this study; Table S5B. Description of the length and function of the identified proteins in this study; Table S5C. List of Non-identified Proteins in this study; Table S5D.List of cello Subcellular localization of novel proteins; Table S5E.List of cello Subcellular localization of identified proteins; Table S5F.List of cello Subcellular localization of unidentified proteins; Table S6: List of conservation analysis of *Nelumbo nucifera* genes in *Arabidopsis lyrata* (A), *Vitis vinifera* (B), *Zea mays* (C), *Glycine max* (D), *Eucalyptus grandis* (E), *Populus trichocarpa* (F), *Prunnus persica* (G), *Glycine soja* (H), *Helianthus annuus* (I), *Juglans regia* (J), *Solanum tuberosum* (K), *Zostera marina* (L), *Oryza sativa* (M), *Musa nana* (N), *Elaeis guineensis* (O), *Daucus carota* (P), *Coffea Arabica* (Q), *Cucumis melo* (R), *Citrus sinensis* (S), *Beta vulgaris* (T), *Amborella trieopoda* (U), *Nymphaea tetragona* (V), *Actinidia chinensis* (W), *Gossypium tomentosum* (X); Table S7: Table S7A. List of biological processes of 4863 identified novel genes; Table S7B. List of molecular functions of 4863 identified novel genes; Table S7C. List of cellular components of 4863 identified novel genes; Table S7D. List of EggNOG function description and COG function classification of 4863 identified novel genes; Table S8: Table S8A. List of 312 novel conservative genes; Table S8B. 110 tissue-specific genes of 312 novel conservative genes; Table S8C. 26 novel tissue-specific genes in C tissue; Table S8D. 18 novel tissue-specific genes in P tissue; Table S8E. 24 novel tissue-specific genes in Cp tissue; Table S8F. 24 novel tissue-specific genes in Sp tissue; Table S8G. 23 novel tissue-specific genes in St tissue; Table S8H.Analysis of conserved domains of 110 novel tissue-specific genes; Table S9: Table S9A.List of all possible novel modifications by MODa; Table S9B.List of 18 common modifications number by MODa.
