# Supplementary material for: Identification of Proteoforms Related to Nelumbo nucifera Flower Petaloid Through Proteogenomic Strategy
Source: Proteomes. 2025 Jan 15;13(1):4. doi: 10.3390/proteomes13010004 (PMC11755666; doi:10.3390/proteomes13010004)
Supplement: Supplementary file 1 [file proteomes-13-00004-s001.zip › proteomes-3249864-supplementary-12.16/Supplementary data/Table S3.pdf]

Table S3 KEGG pathway enrichment analysis of the cor-DEGs-DEPs genes

Pathway	Pathway ID	Geness with pathway annotation
Carbon metabolism	ko01200	51
Citrate cycle （TCA cycle）	ko00020	27
Biosynthesis of amino acids	ko01230	26
Glycolysis / Gluconeogenesis	ko00010	25
Pyruvate metabolism	ko00620	21
Carbon fixation in photosynthetic organisms	ko00710	14
2-Oxocarboxylic acid metabolism	ko01210	12
Ribosome	ko03010	9
Glyoxylate and dicarboxylate metabolism	ko00630	8
Alanine, aspartate and glutamate metabolism	ko00250	8
Peroxisome	ko04146	8
Cysteine and methionine metabolism	ko00270	8
Oxidative phosphorylation	ko00190	8
Propanoate metabolism	ko00640	7
Valine, leucine and isoleucine degradation	ko00280	7
Glutathione metabolism	ko00480	7
Lysine degradation	ko00310	6
Fatty acid degradation	ko00071	6
Tyrosine metabolism	ko00350	6
Pentose phosphate pathway	ko00030	6
Fructose and mannose metabolism	ko00051	6
Glycine, serine and threonine metabolism	ko00260	6
Fatty acid metabolism	ko01212	6
Arginine and proline metabolism	ko00330	6
Starch and sucrose metabolism	ko00500	6
Fatty acid biosynthesis	ko00061	5
beta-Alanine metabolism	ko00410	5
Phagosome	ko04145	5
Pentose and glucuronate interconversions	ko00040	5
Phenylalanine metabolism	ko00360	5
Flavonoid biosynthesis	ko00941	4
Amino sugar and nucleotide sugar metabolism	ko00520	4
Protein processing in endoplasmic reticulum	ko04141	4
Tropane, piperidine and pyridine alkaloid biosynthesis	ko00960	3
Isoquinoline alkaloid biosynthesis	ko00950	3

Table S3 (Continued)

**Pathway**	**Pathway ID**	**Geness with pathway annotation**
Tryptophan metabolism	ko00380	3
Phenylalanine, tyrosine and tryptophan biosynthesis	ko00400	3
Glycerolipid metabolism	ko00561	3
Galactose metabolism	ko00052	3
Phenylpropanoid biosynthesis	ko00940	3
Degradation of aromatic compounds	ko01220	2
Arachidonic acid metabolism	ko00590	2
Biotin metabolism	ko00780	2
Histidine metabolism	ko00340	2
Butanoate metabolism	ko00650	2
Sulfur metabolism	ko00920	2
Pantothenate and CoA biosynthesis	ko00770	2
Biosynthesis of unsaturated fatty acids	ko01040	2
Ascorbate and aldarate metabolism	ko00053	2
Photosynthesis	ko00195	2
Terpenoid backbone biosynthesis	ko00900	2
alpha-Linolenic acid metabolism	ko00592	2
Inositol phosphate metabolism	ko00562	2
Proteasome	ko03050	2
Pyrimidine metabolism	ko00240	2
RNA degradation	ko03018	2
Purine metabolism	ko00230	2
Synthesis and degradation of ketone bodies	ko00072	1
Limonene and pinene degradation	ko00903	1
